# Treating patients suffering from acute coronary syndrome with prasugrel, the Rotterdam experience

**DOI:** 10.1007/s12471-018-1127-z

**Published:** 2018-06-21

**Authors:** R. J. de Winter

**Affiliations:** Academic Medical Center, Amsterdam, The Netherlands

Platelet activation and aggregation that results in intracoronary thrombus formation play a central role in patients who are diagnosed with acute coronary syndrome (ACS). Subsequently, administration of strong platelet inhibitors in the acute phase as well as long-term is one of the mainstays of treatment for ACS. In addition to aspirin, clopidogrel has been recommended after the results of the CURE study were published [1]. And although there was no apparent reduction in mortality, recurrent ischaemic events and stent thrombosis were significantly reduced. Treatment with clopidogrel was not without specific shortcomings, however, with its biological availability dependent on variable intestinal uptake and subsequent conversion to its active metabolite by cytochrome P450 enzymes, who’s effectiveness may be influenced by genotype polymorphisms. Thus, considerable residual on-treatment platelet aggregation or clopidogrel non-responsiveness occurs in a substantial number of patients.

New P2Y12 inhibitors were developed and proofed beneficial in the treatment of ACS compared with clopidogrel, prasugrel in the TRITON TIMI 38 study and ticagrelor in the PLATO study [2, 3]. In TRITON, the design was related to US practice and a percutaneous coronary intervention (PCI) had to be planned, whereas in PLATO a more European approach was adopted and ticagrelor could be administered on admission, also in patients who were managed medically. Tab. 1 shows the hazard ratios and 95% confidence intervals for the ischaemic and bleeding endpoints of both trials.

**Table 1:** Effect of new drugs compared with clopidogrel

	Hazard ratio	Confidence interval
* Prasugrel vs. clopidogrel*		
Urgent target vessel revascularisation	0.66	0.54–0.81
Death from cardiovascular causes	0.89	0.70–1.12
Non-fatal MI	0.76	0.67–0.85
Major TIMI bleeding event	1.32	1.03–1.68
* Ticagrelor vs. clopidogrel*		
Recurrent ischaemia	0.93	0.82–1.05
Death from cardiovascular causes	0.79	0.69–0.91
Non-fatal MI	0.84	0.75–0.95
Major TIMI bleeding event	1.03	0.93–1.15

*MI* myocardial infarction, *TIMI* thrombolysis in myocardial infarction

In a recent meta-analysis, Biondi-Zoccai et al. performed a head-to-head comparison between prasugrel and ticagrelor with the results of the TRITON, PLATO and DISPERSE-2 studies and demonstrated no difference in the risk of overall death, non-fatal myocardial infarction, non-fatal stroke or their composite endpoint [4]. The risk of probable or definite stent thrombosis was lower with prasugrel, at the expense of higher rates of major bleeding events and of bleeding events related to coronary artery bypass grafts (CABG). Current (2015) guidelines of the European Society of Cardiology (ESC) for the treatment of non-ST-elevation ACS have a Class 1 recommendation and level of evidence (LOE) B for both ticagrelor and prasugrel [5]. There is a comment for prasugrel that patients ‘are proceeding to PCI’ thereby limiting the use and timing of prasugrel compared with ticagrelor, which is recommended ‘regardless of initial treatment strategy’ [5]. Clopidogrel received a Class 1/LOE B when prasugrel or ticagrelor are contraindicated or used in combination with oral anticoagulation. Current ESC guidelines for the treatment of ST-elevation myocardial infarction (2017) provide a class I/LOE A recommendation for ticagrelor or prasugrel [6]. The guidelines state that ‘a potent P2Y12 inhibitor (prasugrel or ticagrelor), or clopidogrel if these are not available or are contraindicated, is recommended before (or at latest at the time of) PCI and maintained over 12 months, unless there are contraindications such as excessive risk of bleeding’.

And what about the costs? According to the *Farmacotherapeutisch Kompas*, the annual costs for clopidogrel are € 142.12 (or € 26.97 when generic clopidogrel (€ 0.07/tablet) is given), for prasugrel € 730.87 and for ticagrelor € 877.13 (all including loading doses). In 2016, Wisløff and Atar demonstrated an incremental cost/effectiveness ratio of € 7,700 and € 7,800 per life-year gained for prasugrel and ticagrelor respectively, compared with clopidogrel using data from Norway in a Markov simulation model [7]. Thus, the difference in costs between prasugrel and ticagrelor will probably not be an issue.

In this issue of the journal, Yetgin et al. report the implementation of the guidelines into everyday clinical practice from the Rijnmond Collective Cardiology Research registry [8]. Such registry data provide important additional information compared with the data from highly-selected patients included in randomised clinical trials. Prasugrel was introduced in Rotterdam in August 2011 for the treatment of ACS patients and the data from patients who had been entered into the databases from three PCI centres were collected up to June 2013. In-hospital bleeding events were counted and ischaemic events were followed after hospital discharge. In a total of 2,677 patients, the composite of all-cause death and myocardial infarction at one year occurred in 2.4%, with additional low rates of target vessel revascularisation (3.1%), stent thrombosis (0.6%) and stroke (0.5%). Non-CABG-related thrombolysis in myocardial infarction major bleeding was low with 1.4%. The authors conclude that a tailored approach of prasugrel prescription in ACS patients undergoing PCI resulted in low rates of ischaemic events and bleeding events at one-year follow-up. The authors, together with cardiologists from the PCI and non-PCI centres in Rotterdam, the Netherlands, are to be commended for collecting data from this large patient cohort in everyday clinical practice. We can conclude that treating our ACS patients with prasugrel is safe and effective, provides proven clinical benefit when compared with clopidogrel, and adheres to current European guidelines. For all practical purposes, however, the fact that ‘patients should proceed to PCI’ provides the ‘window of opportunity’ for ticagrelor, which can be given on admission ‘independent of treatment strategy’ [5].
